# Health, Healthcare Access, and Use of Traditional Versus Modern Medicine in Remote Peruvian Amazon Communities: A Descriptive Study of Knowledge, Attitudes, and Practices

**DOI:** 10.4269/ajtmh.14-0536

**Published:** 2015-04-01

**Authors:** Jonathan Williamson, Ronald Ramirez, Tom Wingfield

**Affiliations:** Department of Medicine, University of Bristol Medical School, Bristol, United Kingdom; Amazon Hope Project, The Vine Trust, Iquitos, Peru; Infectious Diseases and Immunity, Imperial College London and Wellcome Trust Imperial College Centre for Global Health Research, London, United Kingdom; The Monsall Infectious Diseases Unit, North Manchester General Hospital, Manchester, United Kingdom

## Abstract

There is an urgent need for healthcare research, funding, and infrastructure in the Peruvian Amazon. We performed a descriptive study of health, health knowledge and practice, and healthcare access of 13 remote communities of the Manatí and Amazon Rivers in northeastern Peru. Eighty-five adults attending a medical boat service were interviewed to collect data on socioeconomic position, health, diagnosed illnesses, pain, healthcare access, and traditional versus modern medicine use. In this setting, poverty and gender inequality were prevalent, and healthcare access was limited by long distances to the health post and long waiting times. There was a high burden of reported pain (mainly head and musculoskeletal) and chronic non-communicable diseases, such as hypertension (19%). Nearly all participants felt that they did not completely understand their diagnosed illnesses and wanted to know more. Participants preferred modern over traditional medicine, predominantly because of mistrust or lack of belief in traditional medicine. Our findings provide novel evidence concerning transitional health beliefs, hidden pain, and chronic non-communicable disease prevalence in marginalized communities of the Peruvian Amazon. Healthcare provision was limited by a breach between health education, knowledge, and access. Additional participatory research with similar rural populations is required to inform regional healthcare policy and decision-making.

## Introduction

The Loreto region of the Peruvian Amazon constitutes only 3.3% of Peru's total population but 28% of Peru's land mass.^1^ One-third of Loreto's population are classified as living in rural subsistence-farming communities, often consisting of both indigenous and mestizo (mixed heritage) people.^2^–^4^ However, Peruvian national health statistics are estimated by geographical area rather than ethnic group and may overlook such communities.^5^ This marginalization may result in inaccurate or absent representation of the healthcare needs of these potentially vulnerable populations.^6^

Nearly 40 years ago, it was noted that the biggest health challenges in Loreto were poverty-related: poverty itself, overcrowding, poor sanitation, and malnutrition.^7^ Despite evidence of some economic development in Loreto since then,^8^ poverty and malnutrition remain common: 42% of the population is classified as living in poverty (living on less than $1.25 US per day), and nearly one-third of children under 5 years old are chronically malnourished.^2^ There are also additional threats to health in the region, with a significant burden of vector-borne diseases, such as malaria^9^ and dengue,^10^ other infectious diseases, such as tuberculosis,^11^ diarrhea (with sporadic cholera outbreaks),^12^,^13^ and retroviral infections, including human immunodeficiency virus^14^ (HIV) and human T lymphotropic virus (HTLV).^15^,^16^

Effectiveness of existing healthcare services in Loreto is restricted because of geographical isolation, limited road access, and underequipped local healthcare services.^9^ Moreover, governmental healthcare initiatives may be biased toward more easily accessible, urbanized regions, such as Loreto's capital Iquitos (population of 500,000).^2^ Non-governmental healthcare provision in the region includes mobile medical boat clinics,^17^ traditional medicine and curanderos (traditional healers),^18^,^19^ or a collision of both modern and traditional practices.^20^ However, there has been minimal research regarding health, health beliefs, and healthcare access of the local communities. To understand and tackle health inequities in Loreto and provide locally and culturally relevant healthcare, additional participatory research that learns from local communities is required.^21^–^23^

During a medical services trip, we performed a descriptive study of health, health knowledge, healthcare access, and traditional and modern medicine practices in remote communities of the Peruvian Amazon.

## Materials and Methods

### Systematic review.

To inform study design and implementation, we searched PubMed and Embase database using the Medical Subject Headings (MeSH) terms “Health behavior[MeSH Terms] OR health knowledge, attitudes, practice[MeSH Terms] OR accessibility, health services[MeSH Terms] AND Perú[MeSH Terms] AND Amazon” with no time or language restriction. Eligible articles had to be a study rather than a review; have health behavior, knowledge, attitudes, practices, or healthcare access as a primary outcome measure; and involve Peruvian Amazon communities.

### Study communities.

The study was undertaken during a medical service trip east of Iquitos, the largest town in Loreto, and visited 13 communities on the Manatí (7 communities) and Amazon (6 communities) Rivers. The medical service provided was externally funded and free of any charges to users for consultations, tests, or prescribed medicines from the boat's on-board pharmacy. This specific medical service has been visiting these 13 communities quarterly for the past 7 years.

### Ethical approval.

The study was approved by the accredited medical ethics committee of the Vine Trust, United Kingdom/Peru. Adults over 15 years of age able to give consent were invited to participate in the study when they came aboard the medical boat clinic for a consultation. Participants were informed that participation would not influence the attention received on the medical boat. All participants gave verbal and written informed consent.

### Structured survey tool.

A locally adapted and appropriate Spanish-language structured survey tool was developed and piloted in the study site (Supplemental Data File). The tool consisted of questions concerning sociodemographic data (including education, crowding, sanitation, and food security); health (including perception and management of pain, diagnosed illnesses, alcohol intake, and smoke exposure); healthcare access and health-seeking behavior (including distance traveled to get to health posts and reasons for not seeking care at the health posts); and knowledge, attitudes, and practices relating to traditional and modern medicine. After consent was given, two Peruvian translators from the region conducted structured interviews with the participants to assist them to fill in the structured survey tool. The translators had a decade of collective experience working with the communities during clinical consultations. Before implementation, the translators received training from the lead author (J.W.) and principal investigator (T.W.) on the content of the tool and how to obtain objective responses without leading or coaching the participants. For privacy and reduced distraction, the structured interview was undertaken away from clinics or waiting areas. In addition to the structured interview aboard the medical boat, four participants who had chronic non-communicable conditions were selected to have a home visit and a more detailed qualitative interview examining traditional versus modern medicine health beliefs and living with chronic disease and disability (reported elsewhere^24^).

### Data analysis.

Categorical data were specified as percentages (*N* = total sample size) and percentages (*n*/*N*) where data were only available for a subset of the population. Sociodemographic, health, healthcare access, and healthcare-seeking data were calculated for the whole cohort and by gender. Means (data with Gaussian distribution), medians (data with non-Gaussian distribution), and ranges were calculated for continuous variables. Participant responses concerning traditional versus modern medicine knowledge, attitudes, and practices were compared using the *Z* test of inference of proportions, which generated 95% confidence intervals (95% CIs) for the responses of these variables. All *P* values were two-sided, and an α-value of 0.05 was taken as the level of significance. All data from questionnaires were checked for validity by first the translator, then the lead author (J.W.), and finally, the principal investigator (T.W.) before they were digitized using Microsoft Excel 2013. Before data transfer to the STATA program (StataCorp, version 12, College Station, TX) for additional analysis, the principal investigator (T.W.) double-checked the data files against the original data sheets to correct any errors.

## Results

### Systematic review.

Our review revealed a paucity of published articles concerning rural health in the Peruvian Amazon. The review found 15 articles, only 3 of which met eligibility criteria. The eligible articles were all survey-based descriptive studies from the Peruvian Amazon: one article concerning disease perception and health-seeking behavior in household contact of tuberculosis patients^25^ and two related papers from 2001^9^ and 2012^26^ describing changes in healthcare access and healthcare behaviors in populations visited during medical service trips. The results of these studies informed our study design and implementation and provided a useful comparison against which to measure our results as described further in Discussion.

### Sociodemographics, poverty, and sanitation.

Sociodemographic data are summarized in Table 1. We surveyed 85 patients, of whom 46 (54%) were male. None of the patients surveyed were from the same household. The average age of the cohort was 40 (18–73) years old, with three children per respondent (0–12 children). The average number of people per house was 5 (1–14 people), with 2 people (0.3–6 people) per room. The majority of respondents had an outdoor latrine (53%), and their principal water supply was from the river (54%). Over one-third of respondents went to bed hungry in the last month because of having no food (35%), and nearly one-quarter had household debt (23%). Despite average respondent age being the same for both sexes, females had a lower educational level (completion of secondary school: 30% versus 50%) and were less likely to be single (18% versus 35%) than males.

### Health, health knowledge, healthcare access, and healthcare-seeking behavior.

Health, health knowledge, healthcare access, and healthcare-seeking behavior data are summarized in Table 2. One-quarter of respondents (25%) had never had a prior formal diagnosis of acute or chronic non-communicable disease or infection. The most common chronic non-communicable disease reported was hypertension (19%). Three participants (4%) reported being patients with diabetes. Of those with a previous diagnosis of a non-communicable disease, 25 (84%) participants reported that they did not completely understand and would like to know more about the diagnosis. The most common acute infection was malaria (13%) followed by renal and/or urinary tract infection (11%). Nearly one-half (48%) of the cohort had consumed alcohol, but only 4% drank one time per week or more. There were 16 (19%) smokers, the majority of whom were men (26% male versus 10% female). The average time taken to get to the nearest health post was 2 hours (range = 1–4 hours), with nearly two-thirds (63%) of participants using a boat as their method of transport. Over two-thirds (68%) of the cohort had previously avoided going to the health post, with the most common reasons for avoidance being distance (47%) and waiting times (32%).

### Pain perception and management.

Participants' perceptions and management of pain are summarized in Table 3. Nearly all (98%) of the cohort experienced pain that was non-traumatic or non-accident–related. The most common cause of pain was musculoskeletal (32%) or head pain (22%). Over one-half (54%) of respondents with pain experienced this pain daily. Seven (9%) respondents described perceived pain that was idiosyncratic or unclassifiable pain, including pain related to high blood sugars or high blood pressure, aura of epileptic or pseudoepileptic seizures, and dizziness interpreted as pain. With regard to gender differences, more women reported musculoskeletal pain or generalized body pain than men (46% versus 20% and 11% versus 0%, respectively). Nearly all respondents reporting pain were able to manage their pain effectively (96%), most commonly with modern medicines/analgesia (72%), but 20% successfully used traditional medicine/analgesia.

### Traditional and modern medicine practices.

**Figure 1.**
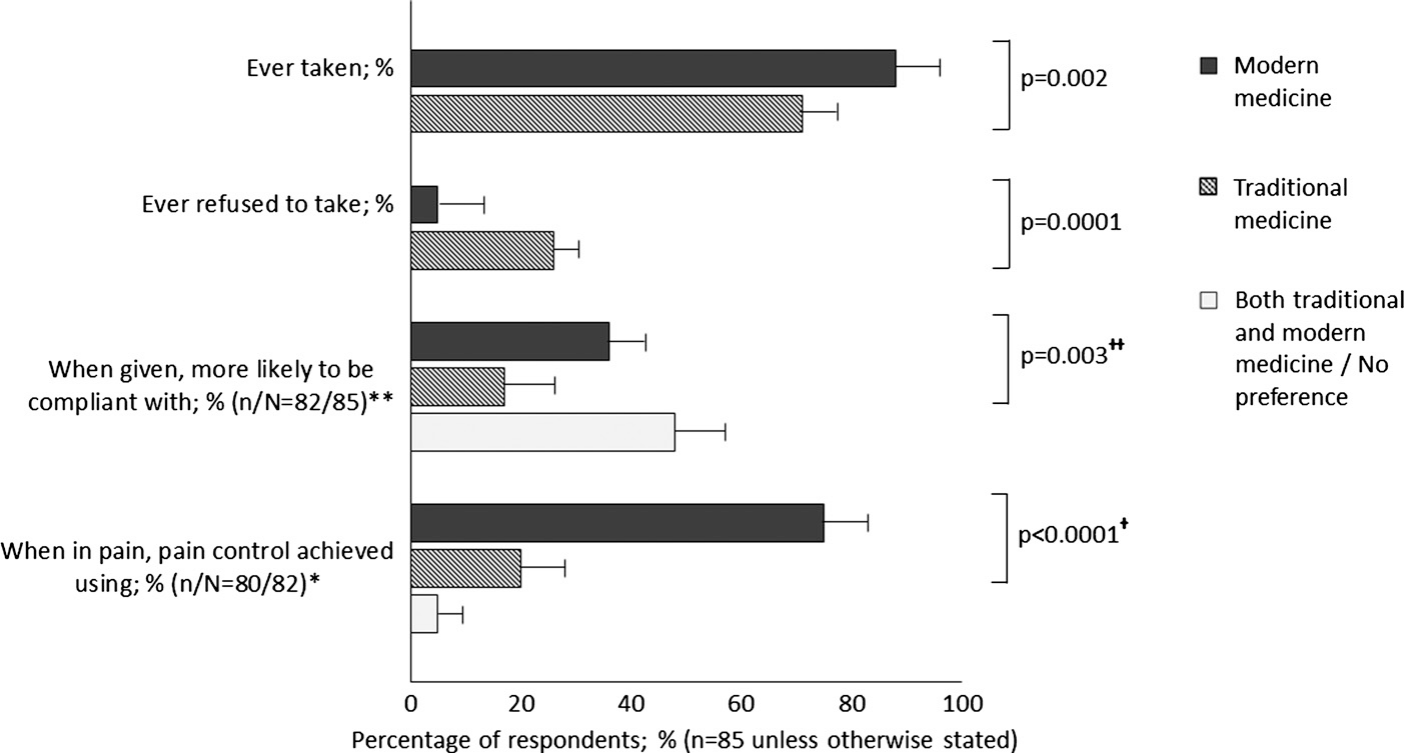
Use of modern and traditional medicine. Note that *P* values relate to the *Z*-test inference of proportions of traditional medicine preference versus modern medicine preference responses. Error bars represent 95% CIs. *n/*N* = 80/82; of 85 participants in the study population, 3 participants did not answer questions regarding pain, and 2 participants were unable to control their pain. **n/*N* = 82/85; three respondents did not answer this question regarding compliance. †The *Z* tests of inference of proportions not shown are modern medicine versus both traditional and modern medicine/no preference (75% [95% CI = 66–84] versus 5% [95% CI = 1–9], *P* < 0.0001) and traditional medicine versus both traditional and modern medicine/no preference (20% [95% CI = 11–29] versus 5% [95% CI = 1–9], *P* = 0.002). ††The *Z* tests of inference of proportions not shown are modern medicine versus both traditional and modern medicine/no preference (36% [95% CI = 29–43] versus 48% [95% CI = 38–58], *P* = 0.1) and traditional medicine versus both traditional and modern medicine/no preference (17% [95% CI = 6–28] versus 48% [95% CI = 38–58], *P* < 0.0001).

Participants' traditional and modern medicine practices are summarized in Figure 1. Participants were more likely to have taken modern medicine than traditional medicine (88% [95% CI = 81–95] versus 71% [95% CI = 61–71], *P* = 0.002) and more likely to have ever refused to take traditional medicine than modern medicine (26% [95% CI = 17–35] versus 5% [95% CI = 1–9]). Of 20 participants who had ever refused to take traditional medicine, 17 (85%) participants cited mistrust and/or lack of belief in traditional medicine, 2 (10%) participants cited side effects, and 1 (5%) participant cited lack of money as the main reason for refusal. Nearly one-half of participants (48%) stated that, if they were prescribed or given traditional or modern medicine, they would be equally compliant with both. Of those who had a preference, more were likely to be compliant with modern rather than traditional medicine (36% [95% CI = 29–43] versus 17% [95% CI = 6–28], *P* < 0.0001). With regard to pain control, participants who suffered with pain were more likely to use modern medicine/analgesia than traditional medicine/analgesia to relieve that pain (75% [95% CI = 66–84] and 20% [95% CI = 11–29], *P* < 0.0001).

## Discussion

This prospective, descriptive study assessed health, health knowledge and practice, and healthcare access in remote Peruvian Amazonian communities receiving care from a mobile medical clinic. We found that poverty and gender inequality were prevalent in this setting. Access to healthcare and healthcare-seeking behavior were limited by long distances to the health post and long waiting times on arrival. Nearly all participants experienced pain on a daily basis, mainly head and musculoskeletal. Non-communicable diseases, such as hypertension, and vector-borne diseases, such as malaria, were common. Nearly all participants felt that they did not completely understand their diagnosed illness and wanted to know more, indicating a breach between health education and knowledge. The preference for modern over traditional medicine use is in contrast to other studies from Peru.^9^,^26^ The novel findings of this study contribute new evidence with which to inform future health policy and planning in the region.

As found in relevant studies in this setting,^9^,^25^,^26^ our results show that poverty indicators, such as household crowding, poor sanitation, and food insecurity, are prevalent. All of these factors have an adverse impact on health: household crowding^27^ and food insecurity^25^ are associated with communicable diseases, such as tuberculosis, and poor sanitation is associated with higher rates of diarrheal disease^13^,^28^ and infant mortality.^13^ Peru has one of the largest indigenous populations in Latin America, most of whom live along the Amazon River and its tributaries.^3^,^4^ In Peru, such rural populations have a disproportionate level of poverty compared with urban populations and may be overlooked and voiceless when it comes to governmental healthcare planning and infrastructure. Over a decade ago, the Peruvian government developed a universal healthcare system (Seguridad Integral de Salud [SIS]) to tackle health disparities. Despite this, there remain people in marginalized and isolated communities of Peru, such as those in the study site, who are unable to access SIS, because they are unsure how to do so or lack the necessary documents (for example, a national identity card).^29^ With regard to sanitation, our findings indicate that the majority of the local population drinks water taken directly from the local rivers. Other studies have also found that the majority of people in this setting drinks unboiled river water, citing boiling water as expensive and time-consuming.^12^,^26^ Such lack of clean drinking water is likely to contribute to rates of water-borne diseases, such as endemic diarrheal disease and sporadic cholera outbreaks.^13^,^14^

The gender differences found in our study are similar to those found by Brierley and others^26^ and Nawaz and others.^9^ Compared with men, women had lower education levels, were less likely to be single, and were more likely to be married. Such gender inequality may relate to sociocultural norms and lower levels of financial independence in women, and it is a contributory factor along the causal pathway of poor health-seeking behavior.^30^ For example, in the Peruvian Amazon, Achuar women are less likely to seek healthcare than men because of language barriers and reluctance to be examined by a male doctor.^5^

Our study provides novel data on non-communicable chronic diseases in this study site. The most common non-communicable chronic disease that the participants—whether male or female—reported to have been diagnosed with was hypertension (19%). Although such hypertension prevalence seems high, there is no previously documented evidence available on hypertension prevalence in this area. However, one other study from a similar but geographically distinct jungle area in central Peru surveyed 76 participants with an average age of 47 years old and found a similar prevalence of 15% hypertension, despite low levels of obesity.^31^ This may indicate that there is a pool of undiagnosed and untreated hypertension in the Peruvian Amazon, perhaps specific to certain ethnic groups. Another possible explanation is that, during medical consultations conducted by T.W. and observed by J.W., patients in the study site commonly attributed their headaches to high blood pressure (alta presión), despite no documented prior diagnosis of high blood pressure.^32^ Other reported chronic non-communicable diseases, such as heart disease and diabetes, were found at levels similar to those found in other rural settings but lower than those found in urban settings.^33^ There is a need to further evaluate these findings with larger prevalence surveys, especially in the face of changing socioeconomic and lifestyle factors leading to transitional communities: increased chronic non-communicable disease morbidity in impoverished communities with high existing burdens of infectious diseases.^33^,^34^

The finding that nearly all of our participants experienced daily pain, mainly headache or musculoskeletal in origin, may be explained by work-related and environmental circumstances. Most families in the study site were subsistence farmers working long hours in their chacras (fields). The physical labor of such agricultural work is demanding, including heavy lifting and maintaining postures that put stress on the spinal column. In addition, the environmental conditions are challenging, with high temperatures, ultraviolet concentrations, and dust levels outside the home and high cooking smoke levels inside the home (especially affecting women and children). Many of the common adult complaints seen aboard the medical service boat related to working conditions: headache secondary to dehydration (it was uncommon to drink water regularly throughout the working day) and sun exposure, cataracts or severe pterygium secondary to sun and dust exposure, back and neck pain related to harvesting and lifting, and skin and soft tissue infections secondary to trauma or water exposure.^17^ To our knowledge, this is the first study to document perceived pain in communities in the Peruvian Amazon, and additional broader research is required to corroborate these findings.

Our results indicate that health knowledge and education were poor. Moreover, there seemed to be a breach between the health education provided by local healthcare services and the perceived knowledge of the local population. These findings elaborate on previous studies from the Peruvian Amazon that suggest poor understanding of endemic diseases, such as malaria^9^,^26^ or tuberculosis,^11^ and are likely to be compounded by the extremely limited access to healthcare services found in this study that typifies the regional situation.

Health education strategies have improved health outcomes, including numbers of soil-transmitted helminth infections in the Peruvian Amazon, although their effect was not long-lasting.^35^ If insensitive to local health beliefs and practices, health education can be a double-edged sword: for example, it has been suggested that increased health promotion activities may be associated with healthcare-seeking delay in tuberculosis patients.^11^ Such adverse outcomes or poor duration of impact of health education may relate to a lack of translation of health care knowledge to practice and behavior.^36^–^38^ For impact to be achievable and durable, locally adapted and culturally appropriate practical health education interventions need to be designed and implemented where need is greatest. However, this is hampered by chronic underfunding, poor staffing, and lack of training of Peruvian healthcare workers on sociocultural health factors.^5^ Local healthcare posts and medical boat services have an opportunity to tackle health promotion through engaging with the local community to discover and dispel health misperceptions; simple educational interventions, such as leaflets or pamphlets; and increasing training of indigenous healthcare workers or promoters.^5^,^9^,^17^

Traditional medical practices in the Peruvian Amazon are ancient and varied from the shamanic practices of curanderos to plant-based natural home remedies (of which one study documented nearly 500 recipes).^39^ Our findings indicate that, although traditional medicine is still prevalent in the communities of the study site, there is a preference for modern medicine, including analgesics. However, there seem to be conflicting findings in the limited literature concerning traditional medicine practice: Nawaz and others^9^ and Brierley and others^26^ found that the majority of respondents at their study sites cited alternative medicine or healers as always or sometimes effective^9^ and traditional medicine to be equal or superior to modern medicine,^26^ whereas other authors describe traditional healers as the primary source of healthcare for the majority of the Amazon region^19^ and traditional medicine use as detracting from uptake of available evidence-based treatments (e.g., antituberculous therapy).^39^ In contrast, our findings and those of other authors suggest that there may be a waning interest in traditional medicine and Penutu (Shaman) practices, which are less highly valued than in the past.^11^,^40^ Potential reasons for this transition may include that, although isolated and remote, our study communities were closer to the largest regional town, Iquitos, than the study sites of Nawaz and others^9^ and Brierley and others^26^ and therefore, had greater exposure to modern medicine and contact with medical boat services; our cohort consisted of patients attending the medical service boat who may have stated a preference for modern medicine, because they believed that this was expected and/or would not pejoratively influence their subsequent consultation; or perhaps, the definitions and practices of traditional medicine are interpreted differently in each community and therefore, not transferable. Additional participatory research^22^,^41^ is required to learn from local indigenous communities' beliefs and thus, potentially incorporate these traditional medicine elements into local healthcare provision alongside modern health promotion and education. For such research to have optimal impact, civil society members, local and indigenous community representatives, and key regional stakeholders should be involved at all stages of the planning and implementation process.

This study has several limitations. First, because of the practical circumstances of conducting a study during a busy medical boat service trip, sampling was non-random, opportunistic, small, and prone to selection bias. The total adult population of the communities visited was approximately 2,400 adults, of whom an average of 720 seek medical attention during each medical services trip to the area. Therefore, our sample of 85 patients represents 12% of all attending adults. It must be acknowledged that people seeking healthcare from a medical boat may represent a different demographic from those remaining in the village, especially with regards to preference for traditional or modern medicine. In addition, there may be seasonal changes in illnesses and health-seeking behavior that could have influenced the study results.^17^ Therefore, the results of the study should be interpreted with caution, because they may not be representative of or generalizable to the wider Amazonian population. Second, for participants whose first language was not Spanish, some of the survey questions may have been difficult to understand. To try to pre-emptively overcome this problem, the lead doctor and coordinator of the medical boat services (R.R.), who is also local to the region, assisted in designing and piloting the study, experienced translators were involved, and daily feedback on questionnaire interpretation took place between the authors (J.W. and T.W.) and translators. Third, it is important to note that we may underestimate the lack of healthcare access in the region: although we describe the study site communities as remote and isolated because of their distance from Iquitos (5–8 hours by the most common form of public boat transport), there are other more distant communities that the medical boat clinic does not reach and therefore, were not studied. In these very remote communities, the health conditions and healthcare access are likely to be even more precarious and limited than the study communities.

There is a pressing need for healthcare research, funding, and infrastructure in the Peruvian Amazon. This study found that healthcare provision in impoverished Peruvian Amazonian communities was limited by lack of access, poor health knowledge, and gender inequality. Our findings also provide new evidence suggesting a hidden burden of pain and chronic non-communicable diseases and a preference for modern over traditional medicine. Furthermore, broader participatory research is required to inform health care policy and decision-making, which are tailored to the needs of the local indigenous populations.

## Supplementary Material

Supplemental Data.

## Figures and Tables

**Table 1:** Socioeconomic demographics of the study population

	Patient *N* (%)	Males *N* (%)	Females *N* (%)
Study population (*N*)	85 (100)	46 (54)	39 (46)
Age (years) median (range)	40 (18–73)	41 (18–73)	40 (19–71)
Number of children (median living and biological; range)	3 (0–12)	3 (0–12)	4 (0–9)
Belong to village distant to Iquitos*	43 (51)	22 (48)	21 (54)
Marital status			
Single	23 (27)	16 (35)	7 (18)
Married	16 (20)	10 (22)	6 (16)
Cohabiting	41 (48)	19 (41)	22 (56)
Widowed	2 (2)	0 (0)	2 (5)
Divorced	1 (1)	1 (2)	0 (0)
Separated	2 (2)	0 (0)	2 (5)
Up to completion of primary school	43 (53)	20 (43)	25 (65)
Up to completion of secondary school	35 (41)	23 (50)	12 (30)
Higher education	5 (6)	3 (7)	2 (5)
People per house median (range)	5 (1–14)	5 (1–14)	6 (1–12)
People per room in house median (range)	2 (0.3–6)	2 (1–6)	2 (1–6)
Toilets (*n*/*N* = 83/85)			
Own facility in the house	10 (12)	4 (9)	6 (16)
Own facility outside of the house	44 (53)	25 (54)	19 (52)
Use public/shared toilet facilities	10 (12)	4 (9)	6 (16)
No toilet facilities	19 (23)	13 (28)	6 (16)
Principal water supply			
Public water supply to tap in house	6 (7)	3 (8)	3 (7)
Well or tank	18 (21)	8 (21)	10 (23)
From river	46 (54)	20 (51)	26 (57)
Rain water	7 (9)	5 (12)	2 (4)
Filtered water (various sources)	7 (9)	3 (8)	4 (9)
Went to bed hungry in the last month because of no food in house	29 (35)	12 (31)	17 (38)
Any formal or informal debt	19 (23)	10 (26)	9 (20)

* Villages along the Manatí Tributary River that were farther from Iquitos than those along the Amazon River.

**Table 2:** Health, healthcare access, and healthcare-seeking behavior

	Patient *N* (%)	Males *N* (%)	Females *N* (%)
Study population (*N*)	85 (100)	46 (54)	39 (46)
Health			
Illnesses			
Never formally diagnosed with an illness/infection	21 (25)	11 (24)	10 (26)
Non-communicable and chronic illnesses (*n*/*N* = 30/85)*			
Hypertension	16 (19)	8 (17)	8 (21)
Joint disease†	6 (7)	3 (7)	3 (8)
Heart disease	4 (5)	1 (2)	3 (8)
Diabetes	3 (4)	2 (4)	1 (3)
Lung disease	3 (4)	3 (7)	0 (0)
Liver and gallbladder disease	3 (4)	0 (0)	3 (8)
Neurological disease	2 (3)	1 (2)	1 (3)
Cancer	1 (1)	0 (0)	1 (3)
Understanding of non-communicable or chronic diagnosed illness (*n*/*N* = 30/85)*			
Understand completely	4 (13)	0 (0)	4 (29)
Partially understand and would like to know more	17 (57)	10 (63)	7 (50)
Partially understand and would not like to know more	0 (0)	0 (0)	0 (0)
Do not understand and would like to know more	8 (27)	5 (31)	3 (21)
Do not understand and would not like to know more	1 (3)	1 (6)	0 (0)
Acute illnesses and infections			
Malaria	11 (13)	7 (15)	4 (10)
Renal and/or urinary tract infection‡	10 (12)	8 (17)	5 (13)
Diarrheal illness	4 (5)	2 (4)	2 (5)
Dengue fever	2 (2)	1 (2)	1 (3)
Tuberculosis	1 (1)	1 (2)	0 (0)
Other illness or infection (chronic or acute)§	13 (15)	8 (17)	5 (13)
Alcohol consumption and smoking history			
Consumes alcohol (*n*/*N* = 84/85)	39 (48)	25 (55)	15 (45)
Consumes alcohol one time per week or more (*n*/*N* = 84/85)	3 (4)	3 (6)	0 (0)
Current cigarette smoker	16 (19)	12 (26)	4 (10)
Number of cigarettes per day (*n*/*N* = 13/16) median (range)	1 (0.1–5)	1 (0.1–5)	2 (1–2)
Healthcare access			
Amazon Hope Medical Boat			
Total number of attendances (*n*/*N* = 84/85) median (range)	4 (1–6)	3 (1–5)	4 (1–6)
Government health posts			
Time (hours) taken to get to health post median (range)	2 (1–4)	2 (1–4)	2 (1–4)
Most likely method of transport to get to health post (*n*/*N* = 84/85)			
On foot	21 (25)	10 (22)	11 (28)
Canoe	9 (11)	5 (11)	4 (10)
Boat¶	53 (63)	29 (64)	24 (62)
Mototaxi	1 (1)	1 (1)	0 (0)
Health-seeking behavior			
Ever avoided going to the health center despite need	57 (68)	35 (61)	22 (56)
Main reason for avoiding the health post (*n*/*N* = 57/85)			
Treated badly by health post staff	1 (2)	0 (0)	1 (5)
Queues/waiting time	18 (32)	9 (26)	9 (41)
Cost	6 (11)	5 (14)	1 (5)
Distance to travel to get to health post	27 (47)	17 (49)	10 (44)
Interfered with work/unable to stop work	5 (8)	4 (11)	1 (5)

* Thirty patients had one or more chronic diagnosed illnesses.

† Joint disease included chronic joint or bone pain for which a doctor had been visited and diagnosed osteoarthritis or rheumatoid arthritis.

‡ It was not possible to separate kidney pain and renal or urinary tract infection as a diagnosis, because in the study setting, the terms are often interchangeable.

§ These were headache (four patients), eye disease (two patients), head injury (one patient), prostatitis (two patients), gastritis (two patients), headache (two patients), unspecified pelvic pain (one patient), and rhinitis (one patient). Two patients experienced combinations of these illnesses or infections.

¶ Larger passenger boat or smaller motorized peque boat.

**Table 3:** Participants' perception and management of pain

	Patient *N* (%)	Males *N* (%)	Females *N* (%)
Study population (*N*)	85 (100)	46 (54)	39 (46)
Perception of pain (*n*/*N* = 84/85)			
Experiences non-trauma or accident-related pain	82 (98)	45 (100)	37 (95)
Part of body experiencing pain (*n*/*N* = 79/82)*			
Musculoskeletal	26 (32)	9 (20)	17 (46)
Head†	18 (22)	9 (20)	9 (24)
Kidney, renal angle, ureters, and bladder	10 (13)	6 (13)	4 (11)
Pelvic‡	10 (13)	5 (11)	5 (14)
Gastrointestinal	9 (11)	3 (7)	6 (16)
Idiosyncratic/unclassifiable§	7 (9)	3 (7)	4 (11)
Chest	5 (6)	4 (9)	1 (3)
Generalized body	4 (5)	0 (0)	4 (11)
Skin	2 (3)	1 (2)	1 (3)
Frequency of pain (*n*/*N* = 79/82)			
Hourly	12 (15)	5 (11)	7 (20)
Daily	43 (54)	25 (57)	18 (51)
Every 2–3 days	11 (14)	5 (11)	6 (17)
Weekly	5 (7)	4 (9)	1 (3)
Every 2–3 weeks	4 (5)	3 (7)	1 (3)
Monthly	4 (5)	2 (6)	2 (5)
Pain management (*n*/*N* = 82/82)			
Able to manage pain effectively	80 (96)	43 (93)	37 (95)
Use of modern medicines/painkillers	60 (72)	30 (65)	30 (81)
Use of traditional medicines/painkillers	16 (19)	9 (20)	7 (19)
Both modern and traditional medicines/painkillers (no preference)	4 (5)	3 (7)	0 (0)

* Certain patients experienced pain in more than one of the parts of the body groupings listed.

† Head pain included headache; eye pain; ear, nose, and throat pain; and maxillofacial pain.

‡ Pelvic pain include unspecified pelvic discomfort, prostate pain in males, and gynecological pain in females.

§ Idiosyncratic/unclassifiable pain was pain that the participant perceived and described that the principal investigator (T.W.) was unable to classify or clinically relate to possible underlying pathology. Examples of such idiosyncratic/unclassifiable pain included pain related to high blood sugars, pain related to high blood pressure, aura of epileptic or pseudoepileptic seizures, and dizziness interpreted as pain.
