# Supplementary material for: Health, Healthcare Access, and Use of Traditional Versus Modern Medicine in Remote Peruvian Amazon Communities: A Descriptive Study of Knowledge, Attitudes, and Practices
Source: Am J Trop Med Hyg. 2015 Apr 1;92(4):857–64. doi: 10.4269/ajtmh.14-0536 (PMC4385786; doi:10.4269/ajtmh.14-0536)
Supplement: Supplementary file 1 [file SD7.pdf]

Supplemental Data File

Contents

Pages 2-7: Spanish Language Questionnaire.
Pages 8-14: Spanish Language Questionnaire with English translation

Spanish Language Questionnaire

Fecha:
Ubicación:

Datos demograficos

Sexo: Varón / Mujer
Edad:
Estado civil: Cual lo describe mejor?

a) Soltero(a)
b) Casado(a)
c) Conviviente
d) Viudo(a)
e) Divorciado(a)
f) Separado(a)

Cuantos hijos tiene? (solo vivos y biológicos) . . . .
Cuanto tiempo lleva viviendo en su domicilio actual?
a) Meses b) Años

Antecedentes

1) Hasta qué grado estudió o estudia usted?

a) Analfabeto
b) Primaria incompleta
c) Primaria completa
d) Secundaria incompleta
e) Secundaria completa
f) Técnica completa
g) Superior completa

2) Alguna vez un doctor le ha dicho que tiene . . . .?

a) Diabetes
b) Enfermedades del corazón
c) Presión alta
d) Cáncer
e) Diarrea (no se necesita un diagnóstico del doctor)
f) Enfermedad (NO doloroso) del riñon
g) Enfermedad del hígado, vesicular
h) Enfermedad neurológica
i) Enfermedades con artritis acompañada por hinchazones y/o deformación de las articulaciones (artritis reumatoide, lupus)
j) Enfermedad respiratoria (embolismo, asma)
k) Otras enfermedades. . . . .(especifique)

Nombre de enfermedad o enfermedades si se sabe.
. . . .
. . . .

3) Si tiene una enfermedad, piensa usted que:

a) Conoce su enfermedad totalmente
b) Conoce su enfermedad parcialmente y querría conocer más sobre la enfermedad
c) Conoce su enfermedad parcialmente y NO querría conocer más sobre la enfermedad
d) NO conoce su enfermedad y querría conocer más sobre la enfermedad
e) NO conoce su enfermedad y NO querría conocer más sobre la enfermedad

Medicamentos

Medicina alternativa o con plantas medicinales

4) Cuando usted se siente enfermo/a, usa usted alguna medicina alternativa / de un curandero?

a) Sí
b) No

**Si se usa medicina alternativa / de curandero, por favor especifique que tipo de medicina / hierba / método de curación usa y para cuales enfermedades suyas?*

....
....

5) Adherencia a medicina alternativa o de curandero (indique o subraye la respuesta que mas le conviene si hay una que le conviene).

a) No toma medicina todos los días porque olvida
b) No toma medicinas todos los días porque aún no está seguro/a porque debe tomarla
c) Dejó de tomar medicina porque "le choca"(porque le hicieron sentirse mal)
d) Dejó de tomar medicina debido al costo
e) Vendió su medicina por cualquier razón
f) Le dió su medicina a un amigo o pariente
g) Siempre

6) Alguna vez ha negado de tomar una medicina sugerida por un curandero o herbero? Si la respuesta es sí, cual fue la razón?

a) Sí
b) No

Especifique la razón...

Medicina moderna o no alternativa

7) Cuando se siente enfermo/a, usa usted alguna medicina moderna?

a) Sí
b) No

*Si es así, se usa medicina moderna o no alternativa, por favor especifique que tipo de medicina / método de curación usa y para cuales enfermedades suyas?

....
....

8) Adherencia a medicina moderna o no alternativa (indique o subraye la respuesta que más le conviene si hay una que le conviene)

a) No toma medicina todos los días porque olvida
b) No toma medicinas todos los días porque aún no está seguro/a porque debe tomarla
c) Dejó de tomar medicina porque le choca (porque le hicieron sentirse mal)
d) Dejó de tomar medicina debido al costo
e) Vendió su medicina por cualquier razón
f) Le dió su medicina a un amigo o pariente
g) Siempre

9) Alguna vez se ha negado a tomar una medicina sugerida por un doctor? Si sí, cual fue la razón?
a) Sí
Especifique la razón...
b) No

Comparación de adherencia de medicinas de distintos tipos

10) Cuál medicina piensa que tomaría tal como le dicen que lo tome?
a) Medicina moderna o no alternativa
b) Medicina alternativa o de curandero o herbero
c) Sería lo mismo para ambos
d) No estoy seguro/a y depende de las circunstancias

Dolor

11) Actualmente o recientemente, sufre o ha estado sufriendo con algún dolor que proviene de una condición diagnosticada por un doctor o no diagnosticada por un doctor?

a) Sí, que proviene de una condición ya diagnosticada
. . . . *(especifique la condición)*
b) Sí, que proviene de una condición no diagnosticada todavía
. . . . (*especifique en que parte del cuerpo le afecta este dolor, por ejemplo las articulaciones, huesos, muslos, la cabeza, el estómago etc*)
c) No

*Si es así, tiene dolor, con qué frecuencia se le ocurre?

a) Cada hora
b) Cada dia
c) Cada 2-3 dias
d) Cada semana
e) Cada 2-3 semanas
f) Cada més

*Puede manejar o superar este dolor efectivamante?

a) Sí, con pastillas anti-inflamatorias modernas
b) Si, con remedios o hierbas naturales . . . . *(especifique el tipo de remedio o hierba)*
c) No, pero el dolor no se me afecta la vida cotidiana
d) No, y el dolor sí se me afecta la vida cotidiana

Servicios de salud/ Health Services

12) Hay algún motivo en especial por el cual usted dude ir al centro de salud?

a) No
b) Mal trato
c) Colas o mucha espera
d) Representa mayores gastos
e) Está lejos
f) Interfiere con el trabajo
g) Otro. . . . .(especifique el motivo)

13) Ha accedido al barco “Amazon Hope” en el pasado?

a) Nunca
b) Sí, pero no este año(por favor eliga cuantas veces ha accedido al barco 1 / 2 /3/ 4/ 5)
c) Sí, una vez este año
d) Sí, dos veces este año
e) Sí, trés veces este año
f) Sí, más de trés veces este año

14) Alguna vez a visitado a un curandero / medico alternativo?

a) Nunca
b) Sí, pero no este año(por favor eliga cuantas veces ha accedido 1 / 2 / 3 / 4 / 5)
c) Sí, una vez este año
d) Sí, dos veces este año e) Sí, trés veces este año
f) Sí, más de trés veces este año

15) Cuanto tiempo demora usted en llegar al puesto de salud más cerca de su casa?

a) Menos de una hora
b) 1-2 horas
c) 2-4 horas
d) Más de 4 horas

16) Qué método de transporte usa para llegar al Puesto de salud? *(especifique, por ejemplo, a pie, barco, carro, mototaxi, una combinada)?*

. . . .

La vivienda

17) Cuantos miembros tiene la familia en total en la actualidad?. . . . . .personas
18) Cuántos miembros de la familia tienen 18 años de edad ó menos en la actualidad?. . . . . .personas
19) Cuantas personas normalmente duermen en casa?. . . . . .personas
20) En cuantas habitaciones duermen estas personas?. . . . . .habitaciones
21) Como se abastece de agua su hogar?

a) Red pública dentro de vivienda
b) Pozo
c) Directamente del río
d) Camión tanque, aguatero
e) Otro. . . . .*(especifique como se abastece)*
f) No hay agua en casa

22) Qué tipo de servicio higienico tiene?

a) Baños públicos
b) Water o baño con taza en la casa
c) Letrina
d) Pozo ciego, silo
e) No tiene servicio higienico
f) Otro. . . . .*(especifique el tipo de servicio)*

23) Cuantas veces en el último més a usted se ha acostado con hambre porque no había comida en casa? . . . . .veces

24) Con qué frecuencia toma usted licor / cerveza / vino o cualquier otro tipo de alcohol?

a) Cada día
b) Cada semana
c) Cada més
d) Cada año
e) Nunca

25) Fuma usted?

a) Sí
b) No

26) Si es así, cuantos cigarillos fuma usted por día? . . . . cigarillos

27) Está usted o su familia endeudada?

a) Sí
b) No

********** **FIN DE ENCUESTA, GRACIAS POR SU VOLUNTAD*************

Questionnaire translated into English

Fecha / Date:
Ubicacion / Location:

Datos demograficos

Sexo / Sex:
Edad / Age:
Estado civil: Cual lo describe mejor? / Marital status: What describes you best?

a) Soltero(a) / Single
b) Casado(a) / Married
c) Conviviente / Cohabiting
d) Viudo(a) / Widow or widower
e) Divorciado(a) / Divorced
f) Separado(a) / Separated

Cuantos hijos tiene? (solo vivos y biologicos) / How many children do you have? (only living or biological)
. . . .

Cuanto tiempo lleva viviendo en su domicilio actual? / How long have you lived at your current address?

Meses / Months   Años / Years

Antecedentes

Hasta qué grado estudió o estudia usted? To what level did you study?

a) Analfabeto / Illiterate
b) Primaria incompleta
c) Primaria completa / Primary school completed
d) Secundaria incompleta
e) Secundaria completa
f) Técnica completa / Secondary school complete
g) Superior completa / Higher degree complete

Alguna vez un doctor le ha dicho que tiene . . . . ?

a) Diabetes / diabetes
b) Enfermedades del Corazon / Heart disease
c) Presión Alta / High blood pressure
d) Cáncer / cancer
e) Diarrea (no necesita un diagnostico medico) / Diarrhoea (doesn't need medical diagnosis)
f) Enfermedad (NO dolor) del riñon / Kidney disease
g) Enfermedad del higado, vesicular / Liver, gallbladder disease
h) Enfermedad neurológica / Neurological condition
i) Enfermedades con artritis acompañada por hinchazones y/o deformación de las articulaciones (artritis reumatoide, lupus) / Autoimmune disease (rheumatoid arthritis / lupus). Enfermedad respiratoria (embolismo, asma) / Lung disease (embolism, asthma)
j) Otras enfermedades (esp) / Other diseases (specify)

Nombre de enfermedad o enfermedades si se sabe / Name of condition(s) if known
. . . .
. . . .

Si tiene una enfermedad, piensa usted que: / If you have a medical condition do you feel you:

a) Conoce su enfermedad totalmente / Understand your disease fully
b) Conoce su enfermedad parcialmente y querría conocer más sobre la enfermedad / Partially understand your disease and WOULD like to know more about it
c) Conoce su enfermedad parcialmente y NO querría conocer más sobre la enfermedad / Partially understand your disease and WOULDN'T wish know more about it
d) NO conoce su enfermedad y querría conocer más sobre la enfermedad / Don't understand your disease and WOULD like to know more about it
e) NO conoce su enfermedad y NO querría conocer más sobre la enfermedad Don't understand your disease and WOULDN'T like to know more about it

Medicamentos / Medicating

Medicina alternativa o con plantas medicinales / Non-Western Medicine

Cuando usted se siente enfermo/a, usa usted alguna medicina alternativa / de un curandero /? / Do you use any non-western medicine, therapy or healing method for illness?

a) Sí / Yes
b) No / No

Si se usa medicina alternativa / de curandero, por favor especifique que tipo de medicina / hierba / método de curación usa y para cuales enfermedades suyas? / If yes please specify medicine, therapy type or healing method and what you use it for.
....

Adherencia a medicina alternativa o de curandero (indique o subraye la respuesta que mas le conviene si hay una que le conviene) / Compliance (select option within answer if there is one)

a) No toma medicina todos los días porque olvida / You don't take medication all the time/none of the time because you forget
b) No toma medicinas todos los días porque aún no está seguro/a porque debe tomarla / You don't take the medication all the time/none of the time because you are unsure why you are taking it
c) Dejó de tomar medicina debido a los efectos adversos o "le choca" (porque le hicieron sentirse mal) / You have stopped taking medication because of the side effects
d) Dejó de tomar medicina debido al costo / You have stopped taking medication because of cost
e) Vendió su medicina por cualquier razón / You have sold on your medication for any reason
f) Le dió su medicina a un amigo o pariente / Given your medication to a friend or relative
g) Siempre/ always adhere

Alguna vez ha negado de tomar una medicina sugerida por un curandero o herbero? Si la respuesta es sí, cual fue la razón? / Have you ever turned down taking a suggested non-western medicine? If so why?

a) Sí / Yes
b) No / No

Especifique la razón...

Medicina moderna o no alternativa / Non-alternative medicine

Cuando se siente enfermo/a, usa usted alguna medicina moderna? / Do you use any non-alternative medicine, therapy or healing method for illness?

a) Sí / Yes
b) No / No

Si sí, se usa medicina moderna o no alternativa, por favor especifique que tipo de medicina / método de curación usa y para cuales enfermedades suyas? / If yes please specify medicine, therapy type or healing method and what you use it for?
....

Adherencia a medicina moderna o no alternativa (indique o subraye la respuesta que mas le conviene si hay una que le conviene) / Compliance (select option within answer if there is one)

a) No toma medicina todos los días porque olvida / You don't take medication all the time/none of the time because you forget
b) No toma medicinas todos los días porque aún no está seguro/a porque debe tomarla / You don't take the medication all the time/none of the time because you are unsure why you are taking it
c) Dejó de tomar medicina debido a los efectos adversos (porque le hicieron sentirse mal) / You have stopped taking medication because of the side effects
d) Dejó de tomar medicina debido al costo / You have stopped taking medication because of cost
e) Vendió su medicina por cualquier razón / You have sold on your medication for any reason
f) Le dió su medicina a un amigo o pariente / Given your medication to a friend or relative
g) Siempre/ Always Adhere

Alguna vez se ha negado a tomar una medicina sugerida por un medico? Si es así, cual fue la razón? / Have you ever turned down taking a suggested modern medicine? If so why?

a) Sí / Yes
b) No / No
Especifique la razón. . . . .

Comparacion de adherencia de medicinas de distintos tipos / Comparison of medicine
Cuál medicina piensa que tomaría tal como le dicen que lo tome? / Which medicine do you think you would be more compliant taking?

a) Medicina moderna o no alternativa / Western medicine
b) Medicina alternativa o de curandero o hebero / Non-Western Medicine
c) Sería lo mismo para ambos / The same level of compliance
d) No estoy seguro/a y depende de las circunstancias / Unsure/depends on other circumstances

Dolor / Pain

Actualmente o recientement, sufre o ha estado sufriendo con algún dolor que proviene de una condición diagnosticada por un doctor o no diagnosticada por un doctor? / Are you currently or have you recently been suffering pain from a known (diagnosed by doctor) or unknown condition?

a) Sí, que proviene de una condición ya diagnosticada / Yes from known condition . . . .(especifique la condición / specify the condition)
b) Sí, que proviene de una condición no diagnosticada todavía / Yes from unknown . . . .(especifique en que parte del cuerpo le afecta este dolor, por ejemplo las junturas, los huesos, los musculos, la cabeza, el estómago etc / specify pain location, e.g joint, muscle, stomach)
c) No / No

Si es así, tiene dolor?, con qué frecuencia se le ocurre? / If yes to the above question. How frequently does this pain occur?

a) Cada hora / Hourly
b) Cada dia / Daily
c) Cada 2–3 dias / Every few days
d) Cada semana / Every week
e) Cada 2–3 semanas / Every few weeks
f) Cada mes / Every month

Puede manejar o superar este dolor efectivamante? Can you effectively manage this pain?

a) Sí, con pastillas anti-inflamatorias modernas / Yes, with western pain killers
b) Si, con remedios o hierbas naturales / Yes, with herbal/plant remedies . . . . (especifique el tipo de remedio o hierba / specifiy)
c) No, pero el dolor no se me afecta la vida cotidiana / No, but the pain DOES NOT effect my daily life
d) No, y el dolor sí se me afecta la vida cotidiana / No, the pain DOES effect my daily life

Servicios de salud/ Health Services

Hay algún motivo en especial por el cual usted dude ir al centro de salud? / Is there any particular reason why you hesítate to go to the health centre?

a) No / No
b) Mal trato / Being poorly treated
c) Colas o mucha espera / Queues or long waits
d) Representa mayores gastos / Represents greater costs
e) Esta lejos / It's far away
f) Interfiere con el trabajo / It interferes with work
g) Otro (esp) / Other (specify)

Ha accedido al barco "Amazon Hope" en el pasado? / Have you visited a Vine Trust medical boat before?

a) Nunca / Never before
b) Sí, pero no este año (por favor eliga cuantas veces ha accedido al barco 1 / 2 / 3 / 4 / 5) Yes but not this year (please circle 1/2/3/4/5/more times)
c) Sí, una vez este año / Once this year
d) Sí, dos veces este año / Twice this year
e) Sí, trés veces este año / Three times this year
f) Sí, más de trés veces este año / More than Three times

Alguna vez a visitado a un curandero / medico alternativo? Have you visited a shaman before?

a) Nunca / Never before
b) Sí, pero no este año (por favor eliga cuantas veces ha accedido 1 / 2 / 3 / 4 / 5) Yes but not this year (please circle 1/2/3/4/5/ more times)
c) Sí, una vez este año / Once this year
d) Sí, dos veces este año / Twice this year
e) Sí, trés veces este año / Three times this year
f) Sí, más de trés veces este año / More than Three times

Cuanto tiempo demora usted en llegar al puesto de salud mas cerca de su casa / How far do you have to travel to your nearest western medicine health post?

a) Menos de una hora / Less than 1 hour
b) 1–2 horas / 1–2 Hours
c) 2–4 horas / 2–4 Hours
d) Más de 4 horas / More than 4 hours

Qué método de transporte usa para llegar al Puesto de salud? (especifique, por ejemplo, a pie, barco, carro, mototaxi, una combinada) / What form of transport do you use to get to the healthpost?
....
....

General Living

Cuantos miembros tiene la familia en total en la actualidad? / How many people make up the family at present?..................................personas

Cuántos miembros de la familia tienen 18 años de edad ó menos en la actualidad? / How many members of the family are less than 18 years of age?.....................personas

How many of the family do NOT work?.....................personas

Cuantas personas normalmente duermen en casa? / How many people normally sleep in the house?.....................personas

En cuantas habitaciones duermen estas personas? / Over how many rooms do these people sleep?.....................habitaciones

Como se abastece de agua su hogar? / How is water provided in you home?

a) Red publica dentro de vivienda / Public water supply in locality
b) Pozo / Water well
c) Directamente del rio / Directly from the river
d) Camion tanque, aguatero / Mobile water supply
e) Otro (esp) / Other (specify)
f) No hay agua en casa / there is no water at home

Que tipo de servicio higienico tiene? / What type of bathroom do you have?

a) Baños públicos / Public toilets in locality
b) Water o baño con taza en la casa
c) Letrina / latrine
d) Pozo ciego, silo / Outdoor hole-in-ground type
e) No tiene / No toilet
f) Otro (esp) / Other (specify)

Cuantas veces en el ultimo mes a usted se ha acostado con hambre porque no habia comida en casa? / How many times in the past month have you gone to bed hungry due to lack of food in the house?..veces

Con qué frecuencia toma usted licor / cerveza / vino o cualquier otro tipo de alcohol? / How often do you drink alcohol?

a) Cada día / each day
b) Cada semana / Weekly
c) Cada més / Monthly
d) Cada año / Yearly
e) Nunca/ never

Fuma usted? / Do you smoke? Sí / Yes No / No

Si es asi, cuantos cigarillos fuma usted por dia: / If you smoke, how many cigarettes do you smoke per day?..................cigarillos

Está usted o su familia endeudada? Are you or your family in debt?

Sí / Yes No / No

********** FIN DE ENCUESTA / END OF QUESTIONNAIRE************
